# Global impact of COVID-19 on stroke care

**DOI:** 10.1177/1747493021991652

**Published:** 2021-07

**Authors:** Raul G. Nogueira, Mohamad Abdalkader, Muhammed M. Qureshi, Michael R. Frankel, Ossama Yassin Mansour, Hiroshi Yamagami, Zhongming Qiu, Mehdi Farhoudi, James E. Siegler, Shadi Yaghi, Eytan Raz, Nobuyuki Sakai, Nobuyuki Ohara, Michel Piotin, Laura Mechtouff, Omer Eker, Vanessa Chalumeau, Timothy J. Kleinig, Raoul Pop, Jianmin Liu, Hugh S. Winters, Xianjin Shang, Alejandro Rodriguez Vasquez, Jordi Blasco, Juan F. Arenillas, Mario Martinez-Galdamez, Alex Brehm, Marios-Nikos Psychogios, Pedro Lylyk, Diogo C. Haussen, Alhamza R. Al-Bayati, Mahmoud H. Mohammaden, Luísa Fonseca, M Luís Silva, Francisco Montalverne, Leonardo Renieri, Salvatore Mangiafico, Urs Fischer, Jan Gralla, ,Donald Frei, Chandril Chugh, Brijesh P. Mehta, Simon Nagel, Markus Mohlenbruch, Santiago Ortega-Gutierrez, Mudassir Farooqui, Ameer E. Hassan, Allan Taylor, Bertrand Lapergue, Arturo Consoli, Bruce CV Campbell, Malveeka Sharma, Melanie Walker, Noel Van Horn, Jens Fiehler, Huy Thang Nguyen, Quoc T. Nguyen, Daisuke Watanabe, Hao Zhang, Huynh V. Le, Viet Q. Nguyen, Ruchir Shah, Thomas Devlin, Priyank Khandelwal, Italo Linfante, Wazim Izzath, Pablo M. Lavados, Veronica V. Olavarría, Gisele Sampaio Silva, Anna Verena de Carvalho Sousa, Jawad Kirmani, Martin Bendszus, Tatsuo Amano, Ryoo Yamamoto, Ryosuke Doijiri, Naoki Tokuda, Takehiro Yamada, Tadashi Terasaki, Yukako Yazawa, Jane G. Morris, Emma Griffin, John Thornton, Pascale Lavoie, Charles Matouk, Michael D. Hill, Andrew M. Demchuk, Monika Killer-Oberpfalzer, Fadi Nahab, Dorothea Altschul, Anna Ramos-Pachón, Natalia Pérez de la Ossa, Raghid Kikano, William Boisseau, Gregory Walker, Steve M. Cordina, Ajit Puri, Anna Luisa Kuhn, Dheeraj Gandhi, Pankajavalli Ramakrishnan, Roberta Novakovic-White, Alex Chebl, Odysseas Kargiotis, Alexandra Czap, Alicia Zha, Hesham E. Masoud, Carlos Lopez, David Ozretic, Fawaz Al-Mufti, Wenjie Zie, Zhenhui Duan, Zhengzhou Yuan, Wenguo Huang, Yonggang Hao, Jun Luo, Vladimir Kalousek, Romain Bourcier, Romain Guile, Steven Hetts, Hosam M. Al-Jehani, Adel AlHazzani, Elyar Sadeghi-Hokmabadi, Mohamed Teleb, Jeremy Payne, Jin Soo Lee, Ji Man Hong, Sung-Il Sohn, Yang-ha Hwang, Dong Hoon Shin, Hong Gee Roh, Randy Edgell, Rakesh Khatri, Ainsley Smith, Amer Malik, David Liebeskind, Nabeel Herial, Pascal Jabbour, Pedro Magalhaes, Atilla Ozcan Ozdemir, Ozlem Aykac, Takeshi Uwatoko, Tomohisa Dembo, Hisao Shimizu, Yuri Sugiura, Fumio Miyashita, Hiroki Fukuda, Kosuke Miyake, Junsuke Shimbo, Yusuke Sugimura, Andre Beer-Furlan, Krishna Joshi, Luciana Catanese, Daniel Giansante Abud, Octavio Giansante Neto, Masoud Mehrpour, Amal Al Hashmi, Mahar Saqqur, Abdulrahman Mostafa, Johanna T. Fifi, Syed Hussain, Seby John, Rishi Gupta, Rotem Sivan-Hoffmann, Anna Reznik, Achmad Fidaus Sani, Serdar Geyik, Eşref Akıl, Anchalee Churojana, Abdoreza Ghoreishi, Mohammad Saadatnia, Ehsan Sharifipour, Alice Ma, Ken Faulder, Teddy Wu, Lester Leung, Adel Malek, Barbara Voetsch, Ajay Wakhloo, Rodrigo Rivera, Danny Moises Barrientos Iman, Aleksandra Pikula, Vasileios-Arsenios Lioutas, Gotz Thomalla, Lee Birnbaum, Paolo Machi, Gianmarco Bernava, Mollie McDermott, Dawn Kleindorfer, Ken Wong, Mary S. Patterson, Jose Antonio Fiorot, Vikram Huded, William Mack, Matthew Tenser, Clifford Eskey, Sumeet Multani, Michael Kelly, Vallabh Janardhan, Oriana Cornett, Varsha Singh, Yuichi Murayama, Maxim Mokin, Pengfei Yang, Xiaoxi Zhang, Congguo Yin, Hongxing Han, Ya Peng, Wenhuo Chen, Roberto Crosa, Michel Eli Frudit, Jeyaraj D. Pandian, Anirudh Kulkarni, Yoshiki Yagita, Yohei Takenobu, Yuji Matsumaru, Satoshi Yamada, Ryuhei Kono, Takuya Kanamaru, Hidekazu Yamazaki, Manabu Sakaguchi, Kenichi Todo, Nobuaki Yamamoto, Kazutaka Sonoda, Tomoko Yoshida, Hiroyuki Hashimoto, Ichiro Nakahara, Elena Cora, David Volders, Celina Ducroux, Ashkan Shoamanesh, Johanna Ospel, Artem Kaliaev, Saima Ahmed, Umair Rashid, Leticia C. Rebello, Vitor Mendes Pereira, Robert Fahed, Michael Chen, Sunil A Sheth, Lina Palaiodimou, Georgios Tsivgoulis, Ronil Chandra, Feliks Koyfman, Thomas Leung, Houman Khosravani, Sushrut Dharmadhikari, Giovanni Frisullo, Paolo Calabresi, Alexander Tsiskaridze, Nino Lobjanidze, Mikayel Grigoryan, Anna Czlonkowska, Diana Aguiar de Sousa, Jelle Demeestere, Conrad Liang, Navdeep Sangha, Helmi L. Lutsep, Óscar Ayo-Martín, Antonio Cruz-Culebras, Anh D. Tran, Chang Y. Young, Charlotte Cordonnier, Francois Caparros, Maria Alonso De Lecinana, Blanca Fuentes, Dileep Yavagal, Tudor Jovin, Laurent Spelle, Jacques Moret, Pooja Khatri, Osama Zaidat, Jean Raymond, Sheila Martins, Thanh Nguyen

**Affiliations:** 1 Neurology, Grady Memorial Hospital, Emory University, Atlanta, Georgia, USA; 2 Radiology, Boston Medical Center, Boston University School of Medicine, Boston, USA; 3 Radiology, Radiation Oncology, Boston Medical Center, Boston University School of Medicine, Boston, USA; 4 Neurology, Grady Memorial Hospital, Emory University, Atlanta, Georgia, USA; 5 Neurology Department, Stroke and Neurointervention Division, Alexandria University Hospital, Alexandria University, Egypt; 6 Stroke Neurology, National Hospital Organization, Osaka National Hospital, Japan; 7 Neurology, Xinqiao Hospital of the Army Medical University, Chongqing, China; 8 Tabriz University, Iran; 9 Neurology, Cooper Neurological Institute, Cooper University Hospital, Camden, New Jersey, USA; 10 Neurology, Radiology, New York University School of Medicine, New York, USA; 11 Radiology, Neurology, New York University School of Medicine, New York, USA; 12 Neurosurgery, Kobe City Medical Center General Hospital, Kobe, Japan; 13 Neurology, Kobe City Medical Center General Hospital, Kobe, Japan; 14 Fondation Ophtalmologique Adolphe de Rothschild, France; 15 Neurologie, Hospices Civils de Lyon, France; 16 Neuroradiologie, Hospices Civils de Lyon, France; 17 Hôpital Bicetre, Paris, France; 18 Royal Adelaide Hospital, Australia; 19 Hôpitaux Universitaires de Strasbourg, France; 20 Changhai Hospital, Shanghai, China; 21 Royal Prince Alfred Hospital, Sydney, Australia; 22 Yijishan Hospital of Wannan Medical College, China; 23 Neurology, Hospital Clinic de Barcelona, Spain; 24 Interventional Neuroradiology, Hospital Clinic de Barcelona, Spain; 25 Neurology, Hospital Clínico Universitario, Valladolid, Spain; 26 Interventional Neuroradiology, Hospital Clínico Universitario, Valladolid, Spain; 27 University Hospital Basel, Switzerland; 28 Clínica Sagrada Familia, Buenos Aires, Argentina; 29 Neurology, Grady Memorial Hospital, Emory University, Atlanta, Georgia, USA; 30 Stroke, Centro Hospitalar Universitário de São João, Portugal; 31 Neuroradiology, Centro Hospitalar Universitário de São João, Portugal; 32 Hospital Geral de Fortaleza, Brazil; 33 Careggi University Hospital, Florence, Italy; 34 Neurology, University Hospital Bern, Switzerland; 35 Interventional Neuroradiology, University Hospital Bern, Switzerland; 36 Swedish Medical Center, USA; 37 MAX Superspecialty Hospital, India; 38 Memorial Neuroscience Institute, Florida; 39 Neurology, University Hospital Heidelberg, Germany; 40 Neuroradiology, University Hospital Heidelberg, Germany; 41 Neurology, University of Iowa, USA; 42 Neurosciences, Valley Baptist Medical Center, Harlingen, Texas, USA; 43 Neurosurgery, University of Cape Town, South Africa; 44 Neurology, Hôpital Foch, France; 45 Interventional Neuroradiology, Hôpital Foch, France; 46 Royal Melbourne Hospital, Melbourne, Australia; 47 Neurology, University of Washington, Seattle, USA; 48 Neurosurgery, University of Washington, Seattle, USA; 49 Interventional Neuroradiology, Universitätsklinikum Hamburg-Eppendorf, Germany; 50 People’s 115 Hospital, Vietnam; 51 IMS Tokyo-Katsushika General Hospital, Japan; 52 Affiliated Hangzhou First People's Hospital, China; 53 Hue Central Hospital, Vietnam; 54 Erlanger Medical Center, USA; 55 Rutgers University, USA; 56 Miami Cardiac and Vascular Institute, USA; 57 Nottingham University Hospitals, United Kingdom; 58 Clínica Alemana, Universidad del Desarrollo, Chile; 59 Universidade Federal de Sao Paulo Hospital Israelita Albert Einstein, Brazil; 60 Hospital Israelita Albert Einstein, Brazil; 61 Hackensack Meridian Health, New Jersey, USA; 62 Neuroradiology, University Hospital Heidelberg, Germany; 63 Kyorin University, Japan; 64 Yokohama Brain and Spine Center, Japan; 65 Iwate Prefectural Central Hospital, Japan; 66 Japanese Red Cross Kyoto Daiichi Hospital, Japan; 67 Kyoto Second Red Cross Hospital, Japan; 68 Japanese Red Cross Kumamoto Hospital, Japan; 69 Kohnan Hospital, Japan; 70 Neurology, Maine Medical Center, USA; 71 Beaumont Hospital, Dublin, Ireland; 72 Hopital Enfant Jesus, Quebec City, Canada; 73 Yale New Haven Hospital, USA; 74 Neurology, University of Calgary, Canada; 75 University Hospital Salzburg, Austria; 76 Emory University School of Medicine, USA; 77 Valley Hospital, New Jersey, USA; 78 University Hospital Germans Trias i Pujol, Barcelona, Spain; 79 Lau Medical Center, Beirut, Lebanon; 80 CHU Montreal, Canada; 81 University of Ottawa, Canada; 82 University of South Alabama, USA; 83 University of Massachusetts Medical Center, USA; 84 University of Maryland, USA; 85 Riverside Regional Medical Center, Virginia, USA; 86 UT Southwestern, Dallas, Texas, USA; 87 Henry Ford Health System, Detroit, USA; 88 Metropolitan Hospital, Piraeus, Greece; 89 UTHealth McGovern Medical School, Houston, USA; 90 SUNY Upstate Medical University Hospital, USA; 91 University Hospital Centre Zagreb, Croatia; 92 Westchester Medical Center, USA; 93 Xinqiao Hospital of the Army Medical University, China; 94 Wuhan No.1 Hospital, China; 95 Affiliated Hospital of Southwest Medical University, China; 96 Maoming Traditional Chinese Medicine Hospital, China; 97 Shaw Shaw Hospital, China; 98 Mianyang 404 Hospital, China; 99 University Clinical Hospital Center Sestre Milosrdnice, Croatia; 100 CHU Nantes, France; 101 University of California San Francisco, USA; 102 King Fahad Hospital of the University, Saudi Arabia; 103 King Saud University, Saudi Arabia; 104 Tabriz University, Iran; 105 Banner Desert Medical Center, USA; 106 Ajou University Hospital, Korea; 107 Kyemyung University, Korea; 108 Kyungpook National University Hospital, Korea; 109 Gachon University Gil Hospital, Korea; 110 Konkuk University Hospital, Korea; 111 St. Louis University, USA; 112 Texas Tech University, USA; 113 Cooper University Hospital, USA; 114 University of Miami, USA; 115 UCLA, Los Angeles, USA; 116 Thomas Jefferson University Hospital, USA; 117 Hospital Sao Jose, Brazil; 118 Eskisehir Osmangazi University, Turkey; 119 Saga-ken Medical Centre Koseikan, Japan; 120 Saitama Medical Center, Japan; 121 Nara City Hospital, Japan; 122 Toyonaka Municipal Hospital, Japan; 123 Kagoshima City Hospital, Japan; 124 Japanese Red Cross Matsue Hospital, Japan; 125 Shiroyama Hospital, Japan; 126 Niigata City General Hospital, Japan; 127 Sugimura Hospital, Kumamoto, Japan; 128 Rush University Medical Center, USA; 129 Neurology, McMaster University, Canada; 130 Interventional Neuroradiology, Ribeirão Preto Medical School, Brazil; 131 Neurosciences, Ribeirão Preto Medical School, Brazil; 132 Shahid Beheshti University, Iran; 133 Khoula Hospital, Ministry of Health, Oman; 134 Hamad Medical Corporation, Qatar; 135 Alexandria University Hospital, Egypt Hamad Medical Corporation, Qatar; 136 Mount Sinai Health System, New York, USA; 137 Cleveland Clinic Abu Dhabi, UAE; 138 WellStar Health, Marietta, Georgia, USA; 139 Rambam Health Care, Israel; 140 General Hospital Dr. Soetomo, Indonesia; 141 Istanbul Aydın University, Turkey; 142 Siriraj Hospital, Thailand; 143 Zanjan University, Iran; 144 Isfahan University, Iran; 145 Qom University, Iran; 146 Royal North Shore Hospital, Australia; 147 Christchurch Hospital, Christchurch , New Zealand; 148 Neurology, Tufts Medical Center, USA; 149 Neurosurgery, Tufts Medical Center, USA; 150 Neurology, Beth Israel Lahey Health, USA; 151 Interventional Neuroradiology, Beth Israel Lahey Health, USA; 152 Neuroradiology, Instituto de Neurocirugia Dr. Asengo, Chile; 153 National Institute of Neurological Sciences of Lima, Peru; 154 University of Toronto, Canada; 155 Neurology, Beth Israel Lahey Health, USA; 156 Neurology, Universitätsklinikum Hamburg-Eppendorf, Germany; 157 University of Texas San Antonio, USA; 158 University Hospitals of Geneva, Switzerland; 159 University of Michigan, USA; 160 Royal London Hospital, United Kingdom; 161 Bon Secours Mercy Health, USA; 162 Hospital-Estadual Central, Brazil; 163 NH Mazumdar Shaw Medical Center, India; 164 University of Southern California, USA; 165 Dartmouth Hitchcock Medical Center, Lebanon, New Hampshire, USA; 166 Neurology, Bayhealth Medical Center, Delaware, USA; 167 Neurosurgery, University of Saskatchewan, Canada; 168 Medical City Plano Texas, USA; 169 St. Joseph’s University Medical Center, USA; 170 Jikei University School of Medicine, Japan; 171 University of South Florida, USA; 172 Changhai Hospital, Shanghai, China; 173 Affiliated Hangzhou First People's Hospital, China; 174 Linyi City People Hospital, China; 175 First People's Hospital, China; 176 Zhangzhou Municipal Hospital, China; 177 Centro Endovascular Neurológico Médica, Uruguay; 178 Universidade Federal de Sao Paulo, Brazil; 179 Christian Medical College, India; 180 Kawasaki Medical School, Japan; 181 Osaka Red Cross Hospital, Japan; 182 University of Tsukuba, Japan; 183 Saiseikai Central Hospital, Japan; 184 Kinikyochuo Hospital, Japan; 185 NTT Medical Center, Japan; 186 Yokohama Shintoshi Neurosurgical Hospital, Japan; 187 Osaka General Medical Center, Japan; 188 Osaka University Graduate School of Medicine, Japan; 189 Tokushima University Graduate School of Biomedical Sciences, Japan; 190 Saiseikai Fukuoka General Hospital, Japan; 191 Tane General Hospital, Japan; 192 Osaka Rosai Hospital, Japan; 193 Fujita Health University School of Medicine, Japan; 194 Dalhousie University, Nova Scotia, Canada; 195 CHU Montreal, Montreal, Canada; 196 McMaster University, Canada; 197 University of Calgary, Canada; 198 Radiology, Boston Medical Center, USA; 199 Lahore General Hospital, Pakistan; 200 Hospital Brasilia, Brazil; 201 University of Toronto, Canada; 202 University of Ottawa, Canada; 203 Rush University Medical Center, USA; 204 UTHealth McGovern Medical School, Houston, USA; 205 National & Kapodistrian University of Athens, Greece; 206 Monash Medical Center, Australia; 207 New York-Presbyterian Queens, USA; 208 Prince of Wales Hospital, Hong Kong; 209 Sunnybrook Health Sciences Centre, Canada; 210 Baptist Health, Arkansas, USA; 211 Fondazione Policlinico Universitario A.Gemelli, Italy; 212 Ivane Javakhishvili Tbilisi State University, Georgia; 213 Adventist Health Glendale, USA; 214 Institute Psychiatry and Neurology, Poland; 215 Hospital de Santa Maria, Portugal; 216 Leuven University Hospital, Belgium; 217 Neurointerventional Radiology, Kaiser Permanente, California, USA; 218 Neurology, Kaiser Permanente, California, USA; 219 Oregon University, USA; 220 Complejo Hospitalario Universitario de Albacete, Spain; 221 Hospital Universitario Ramon y Cajal, Unidad de Ictus, Spain; 222 Hue Central Hospital, Vietnam; 223 Asan Medical Center, Korea; 224 CHU de Lille, France; 225 La Paz University Hospital, Madrid, Spain; 226 University of Miami, USA; 227 Cooper University Hospital, USA; 228 Hôpital Bicetre, Paris, France; 229 University of Cincinnati, USA; 230 Bon Secours Mercy Health, Toledo, Ohio, USA; 231 CHU Montreal, Canada; 232 Hospital de Clínicas de Porto Alegre, Brazil; 233 Radiology, Neurology, Boston Medical Center, USA

**Keywords:** COVID-19, stroke care, acute ischemic stroke, mechanical thrombectomy, intracranial hemorrhage, epidemiology

## Abstract

**Background:**

The COVID-19 pandemic led to profound changes in the organization of health care systems worldwide.

**Aims:**

We sought to measure the global impact of the COVID-19 pandemic on the volumes for mechanical thrombectomy, stroke, and intracranial hemorrhage hospitalizations over a three-month period at the height of the pandemic (1 March–31 May 2020) compared with two control three-month periods (immediately preceding and one year prior).

**Methods:**

Retrospective, observational, international study, across 6 continents, 40 countries, and 187 comprehensive stroke centers. The diagnoses were identified by their ICD-10 codes and/or classifications in stroke databases at participating centers.

**Results:**

The hospitalization volumes for any stroke, intracranial hemorrhage, and mechanical thrombectomy were 26,699, 4002, and 5191 in the three months immediately before versus 21,576, 3540, and 4533 during the first three pandemic months, representing declines of 19.2% (95%CI, −19.7 to −18.7), 11.5% (95%CI, −12.6 to −10.6), and 12.7% (95%CI, −13.6 to −11.8), respectively. The decreases were noted across centers with high, mid, and low COVID-19 hospitalization burden, and also across high, mid, and low volume stroke/mechanical thrombectomy centers. High-volume COVID-19 centers (−20.5%) had greater declines in mechanical thrombectomy volumes than mid- (−10.1%) and low-volume (−8.7%) centers (p < 0.0001). There was a 1.5% stroke rate across 54,366 COVID-19 hospitalizations. SARS-CoV-2 infection was noted in 3.9% (784/20,250) of all stroke admissions.

**Conclusion:**

The COVID-19 pandemic was associated with a global decline in the volume of overall stroke hospitalizations, mechanical thrombectomy procedures, and intracranial hemorrhage admission volumes. Despite geographic variations, these volume reductions were observed regardless of COVID-19 hospitalization burden and pre-pandemic stroke/mechanical thrombectomy volumes.

## Introduction

In December 2019, a novel highly pathogenic virus, severe acute respiratory syndrome coronavirus 2 (SARS-CoV-2), caused an infectious disease involving multiple organ systems termed coronavirus disease 2019 (COVID-19). COVID-19 holds a unique balance between high transmissibility and low-to-moderate morbidity and mortality that has led to a nearly universal spread with devastating consequences worldwide. On 11 March 2020, the World Health Organization declared a global pandemic as COVID-19 hospitalizations and emergency medical system activations increased. As a potential consequence of its neurotropism as well as the inflammatory, immunological, and coagulation disorders, COVID-19 has been reported in association with a broad array of neurological disorders including encephalitis, Guillain-Barre syndrome, seizures, ischemic, and hemorrhagic strokes.^1^ Some groups reported an increase in cryptogenic strokes involving young patients with SARS-CoV-2 infection, possibly in association with endothelial inflammation and thrombotic diathesis.^2–7^ Others reported a decline in the rates of stroke hospitalizations and the proportion of patients receiving reperfusion therapies (intravenous thrombolysis (IVT) and/or mechanical thrombectomy (MT)) for acute ischemic stroke (AIS). Notably, many of these studies originated from global epicenters for the pandemic supporting the notion that the indirect or collateral damage of COVID-19 on systems of care has had a greater impact on stroke patients than the viral infection itself.^3,5,8–12^ However, most of these reports were limited to regional or country-specific analyses, and thus, the extent to which the COVID-19 outbreak has impacted global stroke systems of care has not been previously assessed. Importantly, given the profound benefit of MT in AIS, the global public health impact of such declines, if confirmed, adds to the devastation caused by COVID-19.

## Aims and hypotheses

We conducted an international, observational study on the impact of the COVID-19 pandemic on stroke care at the height of the COVID-19 pandemic. Our primary aim was to evaluate the effect of COVID-19 on stroke care as measured by the changes in volumes for overall stroke hospitalizations, ischemic stroke/transient ischemic attacks (TIA) admissions, ICH admissions, and MT procedures across the pre-pandemic and pandemic periods in a multinational pool of comprehensive stroke centers (CSC). The study compared the three initial months of the pandemic (1 March 2020–31 May 2020) with (1) the immediately preceding months (December 2019–February 2020 for overall volume and November 2019–February 2020 for monthly volume) as the primary analysis and (2) the equivalent three months in the previous year (1 March 2019–30 May 2019) as the secondary analysis. The reason for this analytic hierarchy was an a priori expectation that the volumes for both stroke admissions and MT procedures would increase over time due to the growing evidence supporting the broader utilization of MT.^13–15^ While the primary analysis provided a realistic picture of stroke care utilization prior to COVID-19, the secondary analysis allowed for the assessment for potential seasonal variations.^16^

We hypothesized that in the face of the pandemic’s strain on healthcare infrastructure, (1) a reduction in all four aforementioned measurements of stroke care would take place over the pandemic, (2) centers with higher COVID-19 inpatient volumes would report greater decreases in stroke admissions and MT procedure volumes, (3) the degree of decline in stroke admissions and MT procedure volumes would be less profound in high-volume compared to low-volume stroke centers, and (4) a geographic variation would exist in the intensity of decline in stroke care.

## Methods

Data are available upon request to the corresponding author.

### Study design

This was a cross-sectional, observational, retrospective study evaluating monthly and weekly volumes of consecutive patients hospitalized with a diagnosis of COVID-19, stroke, MT, and ICH. The diagnoses were identified by their related ICD-10 codes (primary, secondary, or tertiary discharge codes) and/or classifications in stroke databases at participating centers.

### Setting and participants

Data were collected from collaborators of the Society of Vascular and Interventional Neurology, the Middle East North Africa Stroke and Interventional Neurotherapies Organization, the Japan Society of Vascular and Interventional Neurology, and academic partners from 6 continents, 40 countries, and 187 CSCs. To reduce bias, only centers providing the full dataset required for any given analysis were included in that specific analysis. Centers were screened for potential confounders that could explain unexpected changes in volumes. One center in Vietnam was excluded from the MT secondary analysis due to an abrupt increase in volume attributed to the purchase of automated imaging software. One center in Brazil was excluded from the stroke admission analysis because it became the designated center for stroke patients, resulting in tripling of their volumes.

### Study variables and outcomes measures

The overall and mean monthly volumes for stroke hospitalizations, admissions for ischemic stroke/TIA, and admissions for ICH and MT procedures were compared across the pandemic and pre-pandemic periods for the overall population and across the low, mid, and high volume strata based on mean monthly volume tertiles for COVID-19 hospitalizations (≤10.6 vs. >10.6–103.6 vs. >103.6 COVID-19 admissions/month), stroke admissions (≤46.2 vs. >46.2–78.4 vs. >78.4 stroke admissions/month), and MT interventions (≤4.8 vs. >4.8 to 11.4 vs. >11.4 procedures/month).

### Statistical analysis

We first compared overall hospital volumes for stroke admissions (overall stroke, ischemic, and ICH) and MT procedures between the pre-pandemic and the pandemic period. For this analysis, the percentage change in the number of admissions or procedures between the two time periods was calculated. The three-month pre-pandemic period was restricted to three months before the pandemic (1 December 2019–29 February 2020) to keep it consistent with the three months during the COVID-19 pandemic group (1 March 2020–31 May 2020). The 95% confidence intervals for percentage change were calculated using the Wilson procedure without continuity correction. The analyses were repeated within each tier (low, mid, and high) of centers classified based on COVID-19 hospitalizations, stroke admissions, and MT procedures. The relative percentage change in overall volume between low, mid, and high-volume centers was tested using the *z-*test of proportion. We also looked at relative change in overall volume by continent.

In the second analysis, we compared monthly hospital volumes (admissions or procedures/hospital/month) for our outcome of interests between the pre-pandemic and the pandemic period. For the pre-pandemic period, for each hospital, the monthly hospital volume was calculated from November 2019 to February 2020 and compared to the monthly hospital volume during the pandemic period (1 March 2020–31 May 2020). The data were analyzed in a mixed design using a repeated-measures analysis of variance (PROC MIXED analysis in SAS) to account for the paired data structure and potential covariates. The auto-regressive, compound symmetrical, and unstructured variance-covariance matrix structures were analyzed for the best model determined by Akaike’s Information Criterion. The unstructured matrix was the best fit and used for most analyses. The monthly hospital volume analysis was adjusted for peak COVID-19 volume for each country and the continent. Estimated marginal means were calculated using the LSMEANS statement in PROC MIXED. Similar to the overall volume analysis, monthly volume analysis was repeated within low, mid, and high tier of centers based on their COVID-19 hospitalizations, stroke admissions, and MT procedures as well as by the continent.

Finally, for our secondary objective, we compared the relative change in overall volume and change in monthly hospital volume during the COVID-19 pandemic and corresponding three months from 2019 (1 March 2019–31 May 2019). All data were analyzed using SAS version 9.4 (SAS Institute), and the significance level was set at a p-value of <0.05.

### Funding and ethics

This was an investigator-initiated project with no funding. The first and last authors wrote the first draft of the manuscript with subsequent input of all co-authors. The institutional review boards from the coordinating sites (Emory University and Boston University) considered that the investigators did not have access to protected health information, and thus no IRB oversight was required since the study did not meet the federal description of human subject research. This study is reported in accordance with the Strengthening the Reporting of Observational studies in Epidemiology (STROBE) statement.

## Results

A total of 16,141, 26,699, and 21,576 stroke hospitalizations (overall *n* = 64,416) and 3397, 5191, and 4533 MT procedures (overall *n* = 13,121) were included across the three-month prior year, three-month immediately pre-pandemic, and three-month pandemic periods, respectively.

### Overall stroke hospitalization volumes

In the primary analysis of overall volume, stroke hospitalization volumes were 26,699 admissions in the three months immediately before compared to 21,576 admissions during the pandemic, representing a 19.2% (95%CI, −19.7 to −18.7, *N* = 121 sites) drop, Table 1. The stroke hospitalization decline had a geographic variation: Asia, −20.5% (95%CI, −21.2 to −19.8); North America, −20.6% (95%CI, −21.4 to −19.7); Europe, −11.2% (95%CI, −12.3 to −10.1); South America, −15.9% (95%CI, −17.9 to −14.0); Oceania, −11.6% (95%CI, −14.4 to −9.3); Africa, −48.1% (95%CI, −55.8 to −40.5), Table S1. In an analysis of monthly volume, after adjustment for peak COVID-19 volume by country and continent, the number of hospitalizations for stroke/month/hospital (adjusted mean (SE)) declined from 76.4 (12.3) pre-pandemic to 64.2 (12.0) during the pandemic (p < 0.0001), Table 1.

**Table 1.** Stroke admissions overall and monthly volumes immediately before and during the COVID-19 pandemic

	Overall volume				Monthly volume^a^			
	*N*	*n*1	*n*2	Change	*N*	Immediately before	During COVID-19	p
				% (95%CI)		Adjusted mean (SE)		
Overall	119	26,699	21,576	–19.2 (−19.7 – −18.7)	121	76.4 (12.3)	64.2 (12.0)	<0.0001
Hospital COVID-19 volume^b^								
Low	38	7612	6654	−12.6 (−13.4 – −11.9)	38	62.4 (31.4)	53.9 (30.7)	0.002
Mid	31	7495	6008	−19.8 (−20.8 – −19.0)	34	84.8 (10.5)	71.0 (8.7)	0.002
High	30	7163	5534	−22.7 (−23.7 – −21.8)	33	90.1 (9.8)	72.9 (9.3)	<0.0001
Hospital stroke volume^c^								
Low	40	3536	3003	–15.1 (–16.3 – –13.9)	40	28.7 (2.6)	24.5 (2.5)	<0.0001
Mid	37	6804	5609	–17.6 (–18.5 – –16.7)	40	62.7 (2.7)	53.1 (3.3)	<0.0001
High	37	14,994	12,400	–17.3 (–17.9 – –16.7)	41	134.1 (21.6)	111.6 (20.8)	<0.0001

*N*: number of hospitals; *n*1: number of admissions immediately before COVID-19 pandemic; *n*2: number of admissions during COVID-19 pandemic; CI: confidence interval; SE: standard error.

Note: The *n*1 is based on 3 months before (December 2019–February 2020) COVID-19 pandemic.

a The monthly volume analysis is adjusted for peak COVID-19 volume for each country and the continent.

b p: low vs. mid ≤ 0.0001; low vs. high ≤ 0.0001; mid vs. high ≤ 0.0001.

c p: low vs. mid = 0.001; low vs. high = 0.002; mid vs. high = 0.588.

### Mechanical thrombectomy procedural volumes

MT volume data was represented by 176 centers in the primary analysis with 5191 procedures in the three months immediately preceding compared to 4533 procedures during the first three months of the pandemic, representing a 12.7% (95%CI, −13.6 to −11.8) decline, Table 2. The volume reduction varied: Asia, −9.8% (95%CI, −11.3 to −8.4); North America, −14.5% (95%CI, −16.2 to −12.9); Europe, −14.4% (95%CI, −16.4 to −12.6); South America, −12.4% (95%CI, −19.0 to −7.9), Oceania, −9.4% (95%CI, −13.4 to −6.5); Africa, −21.2% (95%CI, −37.8 to −10.7), Table S2. The adjusted mean (SE) number of MT procedures/month/center decreased from 10.9 (1.3) pre-pandemic to 9.8 (1.3) during the pandemic (p < 0.0001), Table 2. There were 120 centers that reported concomitant monthly data on stroke admission and MT volume. The adjusted mean (SE) monthly proportion of MT relative to stroke admissions remained stable across the pre-pandemic and pandemic periods (17.8 (2.2)% vs. 18.5 (2.2)%, respectively; p = 0.150). This proportional stability in MT performance was consistent across all COVID-19 and MT hospitalization volumes strata, Table S3.

**Table 2.** Mechanical thrombectomy overall and monthly volumes immediately before and during the COVID-19 pandemic

	Overall volume				Monthly volume^a^			
	*N*	*n*1	*n*2	Change	*N*	Immediately before	During COVID-19	p
				% (95%CI)		Adjusted mean (SE)		
Overall	176	5191	4533	−12.7 (−13.6 – −11.8)	173	10.9 (1.3)	9.8 (1.3)	<0.0001
Hospital COVID-19 volume^b^								
Low	44	952	869	−8.7 (−10.7 – −7.1)	44	11.2 (3.6)	10.5 (3.5)	0.044
Mid	45	1370	1232	−10.1 (−11.8 – −8.6)	45	11.7 (1.2)	10.8 (1.2)	0.004
High	45	1602	1273	−20.5 (−22.6 – −18.6)	46	7.8 (2.2)	5.7 (2.2)	<0.0001
Hospital MT volume^c^								
Low	59	459	412	–10.2 (–13.4 – –7.8)	60	2.6 (0.36)	2.3 (0.36)	0.082
Mid	55	1294	1092	–15.6 (–17.7 – –13.7)	55	8.1 (0.46)	7.0 (0.50)	0.0002
High	58	3432	3029	−11.7 (−12.9 – −10.7)	58	18.8 (1.8)	16.8 (1.7)	0.0002

*N*: number of hospitals; *n*1: number of procedures immediately before COVID-19 pandemic; *n*2: number of procedures during COVID-19 pandemic; CI: confidence interval; SE: standard error; MT: mechanical thrombectomy.

The *n*1 is based on three months before (December 2019–February 2020) COVID-19 pandemic.

a The monthly volume analysis is adjusted for peak COVID-19 volume for each country and the continent.

b p: low vs. mid = 0.259; low vs. high ≤ 0.0001; mid vs. high ≤ 0.0001.

c p: low vs. mid = 0.004; low vs. high = 0.345; mid vs. high = 0.0003.

### Ischemic stroke/TIA and intracranial hemorrhage volumes

The ischemic stroke/TIA admission volumes declined from 19,882 to 16,884 patients across the three months preceding versus the pandemic months, corresponding to a 15.1% (95%CI, −15.6 to −14.6, *N* = 113 sites) reduction with an adjusted mean (SE) number of ischemic stroke or TIA/month/center decreasing from 64.3 (6.8) to 55.6 (6.5) across the two epochs (p < 0.0001). Complete results are presented in Table S4.

The ICH admission volumes, submitted by 100 sites, decreased from 4002 to 3540 patients across the three months immediately before versus the pandemic months, representing an 11.5% (95%CI, −12.6 to −10.6) decline with the adjusted mean (SE) number of hospitalizations for ICH/month/center dropping from 13.4 (2.6) to 11.6 (2.6) across the two periods (p < 0.0001), Table S5.

### Changes in stroke care metrics during the pandemic as a function of COVID-19 hospitalization volumes

**Figure 1.**
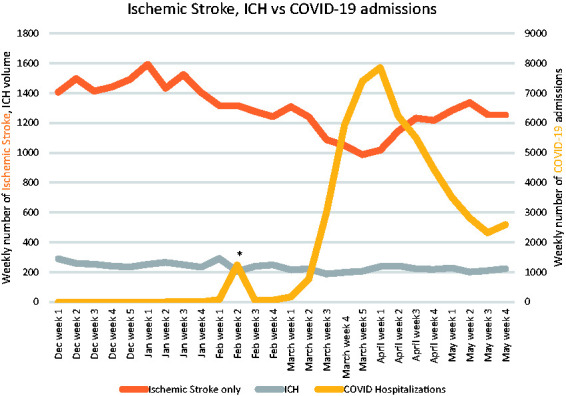
Weekly volume of stroke admissions (ischemic and hemorrhagic) and COVID-19 hospitalizations volumes. *Peak of 1235 COVID hospitalizations in the second week of February, predominantly from one hospital in Wuhan, China.

**Figure 2.**
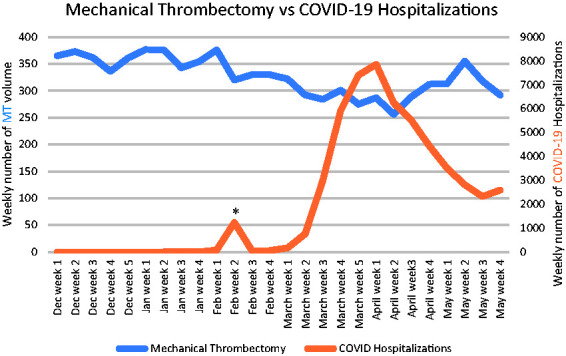
Weekly volume of mechanical thrombectomy and COVID-19 hospitalizations. *Peak of 1235 COVID hospitalizations in the second week of February, predominantly from one hospital in Wuhan, China.

Figures 1 and 2 provide the weekly volume of stroke admissions (ischemic and hemorrhagic), MT, and COVID-19 hospitalizations. COVID-19 hospital weekly volume data was available for 131 centers. There was an early peak of 1235 COVID-19 hospitalizations in February which predominantly originated from one hospital in Wuhan, China. Significant reductions in the mean monthly volumes were seen for all stroke care metrics across all tertiles of low, mid, and high COVID-19 hospitalization volumes. The exception was ICH volumes in high-volume COVID-19 centers which did not show a statistically significant difference (Tables 1, S4, and S5). High-volume COVID-19 centers (−20.5%; 95%CI, −22.6 to −18.6) had greater declines in MT volumes than mid- (−10.1%; 95%CI, −11.8 to −8.6; p < 0.0001) and low-volume (−8.7%; 95%CI, −10.7 to −7.1; p < 0.0001) COVID-19 centers, Table 2. Likewise, high-volume COVID-19 centers (−22.7%; 95%CI, −23.7 to −21.8) had greater reductions in stroke hospitalization volumes than mid- (−19.8%; 95%CI, −20.8 to −19.0; p < 0.0001) and low-volume (−12.6%; 95%CI, −13.4 to −11.9; p < 0.0001) COVID-19 centers, Table 1.

### Changes in stroke care metrics during the pandemic as a function of stroke center MT and admission volumes

Significant declines in the mean monthly volumes were observed for all stroke/MT metrics across low-, mid-, and high-volume stroke/MT centers except MT volumes in low-volume MT centers showed a trend in decline (Tables 1, 2, S4, and S5). Mid-volume stroke centers (−17.6%; 95% CI, −18.5 to −16.7) demonstrated greater decreases in stroke admission volumes than low-volume (−15.1%; 95%CI, −16.3 to −13.9; p < 0.0001) centers, Table 1.

### Secondary objective

Table S6 depicts the volumes for overall stroke, ischemic stroke/TIA, ICH hospitalizations, and MT procedures during the first three months of the pandemic versus the corresponding period in the prior year. Compared to the prior year, there were significant declines in the monthly volumes for stroke and ischemic stroke/TIA admissions but not for ICH and MT.

### Associations between the diagnoses of COVID-19 and stroke

There were 124 centers that reported patients with concomitant stroke (all subtypes) and SARS-CoV-2 infection. To reduce bias, 13 centers with no COVID-19 patients were excluded, leaving 111 eligible centers. A diagnosis of any stroke was present in 791 of 54,366 (1.45%; 95% CI 1.35–1.55) COVID-19 hospitalizations. There was geographic variation with incidences ranging from 0.43% (95%CI 0.08–2.38) in Oceania to 11.9% in South America (95%CI 10.05–14.03), Table S7. Conversely, 784 of the 20,250 (3.9%, 95% CI 3.61–4.14) overall stroke admissions were diagnosed with COVID-19 with proportions varying from 0.14% (95%CI 0.03–0.78) in Oceania to 8.93% in South America (95%CI 7.54–10.55), Table S8.

## Discussion

We noted a significant global decline in all measured stroke care metrics in the current study including the numbers of mechanical thrombectomy procedures (−12.7%), overall stroke admissions (−19.2%), ischemic stroke/TIA admissions (−15.1%), and intracranial hemorrhage hospitalization volumes (−11.5%) during the COVID-19 pandemic as compared to the immediately preceding three months, confirming our primary hypothesis. Volume reductions were also seen in relation to the equivalent period in the prior year for stroke admissions and ischemic/TIA admissions. The intensity of the decline was more pronounced when comparing the pandemic period with the immediate three months prior than with the same months in 2019 (MT: 12.7% vs. 6.0%; stroke admissions: 19.2% vs. 12%). This followed our a priori expectations in face of the expansions in MT indications along with its progressive but gradual global implementation in developed and developing countries.^17^ Interestingly, despite the absolute decrease in MT volumes, the proportion of MT relative to stroke admissions remained stable during the pandemic. While at first glance this might suggest that the intra-hospital workflow was maintained, it is possible that this was not the case since one would actually expect an increase in the MT ratio relative to stroke admissions as many studies have now demonstrated that there was a preferential decline in patients presenting with milder strokes during the pandemic.^4,11,18–20^ The decreases in the amount of stroke care were noted across centers with high, mid, and low COVID-19 hospitalization burden and also across high, mid, and low volume stroke and MT centers. As hypothesized, centers with higher COVID-19 inpatient volumes suffered more declines. Contrary to our expectations, the declines in stroke hospitalizations and MT volumes were more profound in mid-(and high-) volume than low-volume stroke centers. This might be related to the fact that larger centers were more likely to become the preferred destination for COVID-19 referrals leading to capacity issues. Finally, we confirmed a broad geographic variation in the patterns of stroke care decline.

Our results align with recent reports emphasizing the collateral effects of the COVID-19 pandemic on stroke systems of care from China,^10,18^ Spain,^3,19^ Italy,^21,22^ France,^9,23^ Germany,^12^ Brazil,^20^ Canada,^24^ and United States,^5,11,25,26^ showing declines in the volumes for MT, IVT, and stroke hospitalizations over the pandemic (Table S9–11). Some of these studies also reported delays in hospital arrival times^18,21,25^ and treatment workflow.^9,21^ Our analysis adds to the growing literature regarding the collateral damage of COVID-19 on stroke care with the advantage of providing a broader global perspective. While the overall data clearly points to a significant reduction in the quantity of stroke care provided during the pandemic, it also depicts variations within and across the different regions reflecting the diversity in the epidemiology for COVID-19 as well as in the socio-cultural behaviors, healthcare logistics, and infrastructure encountered across the globe. Indeed, our study demonstrated important geographic variations in the proportional declines for both stroke hospitalization and MT volumes. Notably, our analysis may have underestimated the impact of geographic disparities in healthcare resources and related socio-economic factors as we only included thrombectomy capable centers which are known to have better infrastructure than the more commonly found primary stroke centers. Moreover, there was a higher geographic variation in the proportional decline for stroke hospitalization (Asia, −20.5%; North America, −20.6%; Europe, −11.2%; South America, −15.9%, Oceania, −11.6%; Africa, −48.1%) than mechanical thrombectomy (Asia, −9.8%; North America, −14.5%; Europe, −14.4%; South America, −12.4%, Oceania, −9.4%; Africa, −21.2%) volumes. As seen in relation to the stability in the MT ratio relative to stroke admissions, this might have been related to the favored decline in milder strokes over the course of the pandemic.^4,11,18–20^ Given the growing evidence supporting the association between COVID-19 and thromboembolic events, it would be expected that the stroke incidence would rise at the precipice of the pandemic. Several factors may explain this paradoxical global decrease in stroke, MT, and ICH volumes observed in this study. As this decline in stroke volume was seen in centers with low or non-existent COVID-19 hospitalizations, hospital access due to the COVID-19 hospitalization burden was unlikely a major factor.^12^ As elective surgeries were canceled with the pandemic, a decrease in perioperative stroke may have played a role. It is also conceivable that the environmental situation of a lockdown, with improved patient behaviors or medication compliance, may be protective in decreasing vascular events.^27^ A reduction in exposure to other common viruses that may play a role in triggering vascular events may have also reduced stroke risk. However, it is unlikely that true incidence of stroke declined and more likely the behavioral and infrastructural changes related to the pandemic led to a reduction of admission of AIS patients, especially during the initial phases of public lockdown. Fear of contracting SARS-CoV-2 may have led many patients with milder stroke presentations to avoid seeking medical attention.^4,11,18–20^ Physical distancing measures may have prevented patients from the timely witnessing of a stroke.

Our subgroup of 111 centers including 54,366 COVID-19 hospitalizations is the largest sample reporting the concomitant diagnoses of stroke and SARS-CoV-2 infection to date. Our 1.45% stroke rate in COVID-19 hospitalizations is similar to the pooled incidence of 1.1–1.2% (range, 0.9–2.7%) of hospitalized COVID-19 patients.^28,29^ Some variations in the proportions are expected given the different definitions (all strokes vs. ischemic only) and populations involved (all hospitalized vs. severely infected only) across studies. We also provide a new perspective on this relationship by reporting an incidence of 3.9% (784/20,250) for SARS-CoV-2 infection across all stroke admissions among centers with documented COVID-19 hospitalization.

### Study strengths and limitations

The strength of our study was the large volume of patients (*n* = 64,416) and a high number of centers (*n* = 187) contributing data from a diverse population across six continents and 40 countries. Our study contained centers with high and low COVID-19 hospitalization admissions, high and low stroke admission, and MT volumes, permitting the generation of multiple hypotheses and endpoints.

The limitations of this study were that the diagnosis of stroke/TIA/ICH, thrombectomy volume in some centers was obtained using hospital ICD administrative codes, and verification for accurate diagnosis was not universally undertaken. The centers contributing to these data have systems in place to track stroke admissions; thus, the relative changes in volume from this analysis are likely accurate. Details on patient-level data including demographics, stroke subtypes, and clinical outcomes were not collected as these were not the focus of the study. As with any other study, our data may underestimate true rates of concomitant SARS-CoV2 infection with a stroke diagnosis depending on the frequency of testing at each site and across the study period. The definition of the pandemic period was arbitrary since the outbreak started and peaked at different times at different locations. After adjustment for peak COVID-19 volume for each country and continent, the monthly volume declines were retained for all stroke metrics (stroke hospitalization, MT, ICH). As the penetration of MT remains limited in many countries,^17^ some geographic regions were not represented (i.e. central Africa). We did not collect data on the timing or intensity of social distancing policies including lockdown implementation across the different localities which likely played an important role in the reported stroke care decline. Finally, the sampling varied with the availability of complete data in each subset of the analysis.

## Summary

There was a significant global decline in mechanical thrombectomy and stroke admissions over the three months studied during the pandemic. These decreases were seen regardless of COVID-19 admission burden, individual pre-pandemic stroke, and MT volumes. Thus, it is critical to expeditiously raise public awareness to prevent the additional healthcare consequences associated with the lack of stroke treatment. These findings can inform regional stroke networks preparedness^29^ in the face of a future pandemic or anticipated surge of COVID-19 cases in order to ensure that the access and quality of stroke care remains preserved despite the crises imposed by the continuous spread of the virus.

## ORCID iDs

Y Mansour https://orcid.org/0000-0001-8814-6431

Zhongming Qiu https://orcid.org/0000-0002-1622-9526

James E Siegler https://orcid.org/0000-0003-0287-3967

Laura Mechtouff https://orcid.org/0000-0001-9165-5763

Omer Eker https://orcid.org/0000-0002-5696-5368

Raoul Pop https://orcid.org/0000-0003-4417-1496

Juan F Arenillas https://orcid.org/0000-0001-7464-6101

Mahmoud Mohammaden https://orcid.org/0000-0002-7393-9989

Simon Nagel https://orcid.org/0000-0003-2471-6647

Mudassir Farooqui https://orcid.org/0000-0003-3697-5697

Ameer E Hassan https://orcid.org/0000-0002-7148-7616

Allan Taylor https://orcid.org/0000-0002-2692-2068

Bertrand Lapergue https://orcid.org/0000-0002-8993-2175

Bruce CV Campbell https://orcid.org/0000-0003-3632-9433

Noel Van Horn https://orcid.org/0000-0001-5764-1982

Ryoo Yamamoto https://orcid.org/0000-0001-8585-9831

Takehiro Yamada https://orcid.org/0000-0001-5196-1934

Yukako Yazawa https://orcid.org/0000-0003-3891-0221

Jane Morris https://orcid.org/0000-0001-5256-8165

Michael D Hill https://orcid.org/0000-0002-6269-1543

Thanh Nguyen https://orcid.org/0000-0002-2810-1685
